# Clinical and immune profiling for cancer of unknown primary site

**DOI:** 10.1186/s40425-019-0720-z

**Published:** 2019-09-13

**Authors:** Koji Haratani, Hidetoshi Hayashi, Takayuki Takahama, Yasushi Nakamura, Shuta Tomida, Takeshi Yoshida, Yasutaka Chiba, Takahiro Sawada, Kazuko Sakai, Yoshihiko Fujita, Yosuke Togashi, Junko Tanizaki, Hisato Kawakami, Akihiko Ito, Kazuto Nishio, Kazuhiko Nakagawa

**Affiliations:** 1 Department of Medical Oncology, Kindai University Faculty of Medicine, 377-2 Ohno-higashi, Osaka-Sayama, Osaka, 589-8511 Japan; 2 Department of Genome Biology, Kindai University Faculty of Medicine, Osaka-Sayama, Japan; 3 Department of Pathology, Kindai University Faculty of Medicine, Osaka-Sayama, Japan; 4 Department of Biobank, Graduate School of Medicine, Dentistry, and Pharmaceutical Sciences, Okayama University, Okayama, Japan; 5 Clinical Research Center, Kindai University Hospital, Osaka-Sayama, Japan; 6 Kindai University Life Science Research Institute, Osaka-Sayama, Japan; 7 Division of Cancer Immunology, Research Institute/Exploratory Oncology Research and Clinical Trial Center, National Cancer Center, Kashiwa, Japan; 8 Department of Medical Oncology, Kishiwada City Hospital, Kishiwada, Japan

**Keywords:** Cancer of unknown primary site (CUP), Gene expression, Immune profile, Immunotherapy, Immune checkpoint inhibitor

## Abstract

**Background:**

Immune checkpoint inhibitors (ICIs) confer a survival benefit in many cancer types. Given that the survival outcome for cancer of unknown primary site (CUP) remains poor, we investigated the potential of CUP for immunotherapy.

**Methods:**

A total of 164 patients with CUP (favorable subset, 34 patients; unfavorable subset, 130 patients) who were treated between January 2009 and March 2017 was identified from a review of medical records at Kindai University Hospital. They included 92 patients for whom pretreatment tumor tissue was available both for determination of programmed cell death–ligand 1 expression and tumor-infiltrating lymphocyte (TIL) density by immunohistochemistry (IHC) and for immune-related gene expression profiling (irGEP). The results of irGEP for CUP were compared with published data for ICI-treated solid cancers classified into progressive disease (PD) and non-PD subsets according to their best response to ICIs.

**Results:**

The median overall survival of all CUP patients was 29.3 months (95% confidence interval [CI], 15.7–not reached) and 7.1 months (95% CI, 5.0–9.4) for favorable and unfavorable subsets, respectively. IHC and irGEP revealed that pretreatment immune activity—including expression of immune checkpoint molecules—for CUP was similar to that for ICI-responsive malignancies (antitumor immune cell signatures: CUP versus PD, *P* = 0.002–0.067; CUP versus non-PD, *P* = 0.591–0.999), although *VEGFA* expression was associated with suppression of antitumor immunity in CUP (*P* = 0.008, false discovery rate = 0.010). In addition, one case of CUP in the unfavorable subset that was associated with prominent PD-L1 expression on TILs and showed a durable response to nivolumab is presented.

**Conclusions:**

The survival outcome of CUP remains unsatisfactory. However, our clinical and immune profiling of CUP has revealed a potential to benefit from immunotherapy, with ICIs thus being a potential option for CUP treatment.

**Electronic supplementary material:**

The online version of this article (10.1186/s40425-019-0720-z) contains supplementary material, which is available to authorized users.

## Background

Cancer of unknown primary site (CUP) accounts for 2 to 5% of all diagnosed cancers and is associated with poor prognosis [1, 2]. CUP is usually diagnosed after metastasis has occurred, with the anatomic site of the primary tumor not being amenable to identification even after thorough clinical examination. In addition, CUP is clinically heterogeneous as a result of its biological origins including various types of cancer. Given this background, treatment of CUP is problematic and has not been well developed [1, 2].

CUP is divided into favorable and unfavorable subsets according to its clinical presentation, with treatment traditionally having been based on such classification [1, 2]. CUP of the favorable subset is usually treated as are specific cancer types, with these specific cancer type–oriented therapies conferring a better prognosis in the favorable subset relative to that achieved for patients in the unfavorable subset. However, the outcome of such treatment is not satisfactory—with median overall survival (OS) having been reported as only 1 to 3 years—as a result of subsequent recurrence in most cases [1, 2]. Most patients with the unfavorable subset of CUP receive palliative treatment with empirical chemotherapy, although a survival benefit for this approach has not been demonstrated and survival for the unfavorable subset is generally < 1 year [1, 2]. Personalized medicine based on molecular profiling such as gene expression–guided chemotherapy or genome sequence–guided molecular therapy has been developed for the treatment of CUP, but the clinical benefit of such emerging therapies remains unclear [3–5].

The advent of immune checkpoint inhibitors (ICIs) has led to a marked improvement in survival for patients with various types of malignancy, including non–small cell lung cancer (NSCLC), gastroesophageal cancer, genitourinary cancer, and head and neck cancer (HNC) [6]. Postmortem analysis and gene expression profiling have identified these cancer types as common occult origins of CUP [7], suggesting that ICIs might also prove effective for the treatment of CUP [8]. However, little is known about the immunologic suitability of CUP for ICI therapy. Given that, even among patients with cancer types in which ICI therapy has become a standard of care, not all individuals—such as those with insufficient immune infiltration or immune-related gene expression—respond to ICI treatment [9–13], exploratory studies of the immune profile of CUP are necessary before prospective interventional studies with ICIs can be conducted.

We have therefore now performed such an exploratory study to evaluate the immune profile of CUP and its potential suitability for treatment with ICIs. A clinical database was reviewed for CUP patients, and available tumor tissue was analyzed by immunohistochemistry (IHC) and immune-related gene expression profiling (irGEP).

## Methods

### Patients and samples

**Fig. 1:**
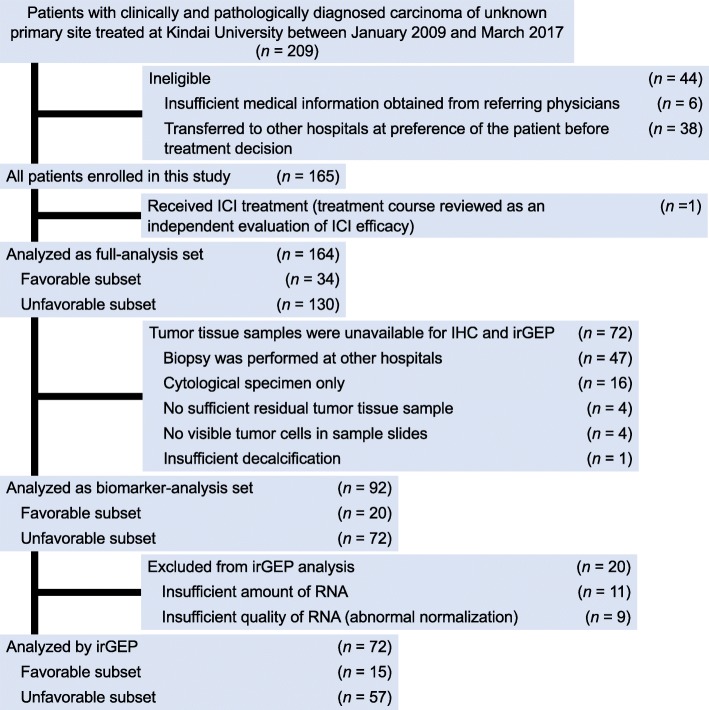
Flow of the study patients with cancer of unknown primary site. ICI, immune checkpoint inhibitor; IHC, immunohistochemistry; irGEP, immune-related gene expression profiling

We reviewed the medical records of all patients with CUP diagnosed at Kindai University Hospital between January 2009 and March 2017. CUP was defined as a pathologically diagnosed carcinoma in a patient presenting with metastatic lesions for which the primary origin was not identifiable unequivocally on the basis of thorough physical examinations including a gynecological workup for females, serum markers, diagnostic imaging, and detailed pathological analysis with IHC when needed. Further clinical examinations such as esophagogastroduodenoscopy, colonoscopy, and breast imaging were also performed at the discretion of the treating physician according to published clinical practice guidelines [1, 2]. ^18^F-fluoro-2-deoxy-d-glucose–based positron emission tomography combined with computed tomography (FDG-PET/CT) was performed in 96% of the cohort. The classification of patients into favorable and unfavorable subsets was based on published clinical practice guidelines [1, 2]. Patients with neuroendocrine carcinoma (NEC), squamous carcinoma limited to cervical lymph nodes (HNC-like), adenocarcinoma restricted to axillary lymph nodes (LNs) in females (breast cancer [BC]–like), extragonadal germ cell tumor syndrome (GCT-like), peritoneal carcinomatosis in females (primary peritoneal cancer [PPC]–like), squamous carcinoma limited to inguinal LNs (anal canal carcinoma [ACC]–like), or single resectable metastatic carcinoma were thus included in the favorable subset. Patients who did not meet these definitions were classified into the unfavorable subset. From this review, we identified 209 CUP patients, of whom 44 were ineligible because of insufficient medical information or treatment history (Fig. 1). Postmortem examination was not performed in this cohort. Among enrolled patients, only one individual received ICI treatment during the study period; this patient was excluded from the main analyses, but her clinical course is presented as an independent evaluation of ICI efficacy. The remaining 164 patients were subjected to clinical profiling as the full-analysis set. In addition, 92 of these patients were included in the biomarker-analysis set because they had pretreatment archival formalin-fixed paraffin-embedded (FFPE) tumor tissue available for IHC and irGEP. Details of patient recruitment are shown in Fig. 1. The study was performed according to the Declaration of Helsinki and protocols approved by the Institutional Review Board and Ethical Committee of Kindai University Faculty of Medicine.

### Data collection

Medical records were reviewed, and data regarding clinicopathologic features and treatment history were extracted. Data were updated as of 30 September 2018. Overall survival (OS) was measured from treatment initiation to death from any cause. Patients who were still alive were censored on the date of last follow-up. All archival tumor tissues for biomarker analyses was collected before any treatment was given, with the exception of one specimen that was obtained after disease progression during platinum-based cytotoxic chemotherapy.

### IHC

Sections of FFPE tumor tissue (thickness, 4 μm) from patients in the biomarker-analysis set were subjected to IHC with the use of an automated stainer (Dako) and with monoclonal antibodies to programmed cell death–ligand 1 (PD-L1) (clone 28–8, Abcam), to CD8 (clone C8/144B, Agilent Technologies), to forkhead box P3 (FOXP3) (clone 236A/E7, Abcam), to lymphocyte activation gene-3 (LAG-3) (clone 17B4, Abcam), and to T cell immunoglobulin and mucin domain-3 (TIM-3) (clone D5D5R, Cell Signaling). The stained slides were evaluated by two independent board-certified pathologists who were blinded to clinical outcome. The percentage of tumor cells positive for PD-L1 was determined as the PD-L1 tumor proportion score (TPS). The combined positive score (CPS) for PD-L1 expression was also calculated as the number of PD-L1–positive cells (tumor cells, lymphocytes, macrophages) divided by the total number of tumor cells and multiplied by 100 [14]. PD-L1 positivity was defined as membranous staining at any intensity [15, 16]. For slides with discrepant scores, the final score was determined after review of the slides and discussion by the two pathologists. Concordance between the two observers was 1.00 (κ = 1.00) for PD-L1 TPS with a cutoff value of ≥1%, and 0.94 (κ = 0.87) for PD-L1 CPS with a cutoff value of ≥1%. Tumor-infiltrating lymphocytes (TILs) were evaluated on the basis of staining for CD8, FOXP3, LAG-3, and TIM-3 [11]. The number of TILs was determined as the absolute count of cells positive for each marker at any staining intensity (CD8^+^ TILs, FOXP3^+^ TILs, LAG-3^+^ TILs, or TIM3^+^ TILs). At least one and a maximum of five fields of tumor regions were randomly chosen for each TIL count. The density of TILs in the tumor was calculated by dividing the number of TILs by the sum of the area (mm^2^) of the viewed fields. TILs were counted independently by the two pathologists, and the average of each count was reported as the final score.

### irGEP

A section of FFPE tumor tissue was first examined by hematoxylin-eosin (HE) staining to confirm the presence of invasive tumor cells and to determine the tumor area. Macrodissection of the tumor lesions was performed before RNA extraction. We excluded specimens with only small undissectable metastatic lesions in LNs so as to avoid contamination by non–tumor-infiltrating immune cells located in the normal LN area (which can lead to overestimation of immune activity). RNA was extracted from the dissected FFPE tumor tissue with the use of an AllPrep DNA/RNA FFPE Kit (Qiagen), and it was concentrated with the use of an RNA Clean & Concentrator (Zymo Research) as needed. The amount of extracted RNA was measured with a NanoDrop system (Thermo Fisher Scientific), and a minimum of 50 ng of total RNA was used for gene expression analysis with the nCounter platform and a PanCancer Immune Profiling Panel comprising 730 immune-related genes and 40 housekeeping genes (NanoString Technologies). Tumor-derived RNA obtained from 81 patients was thus analyzed. In addition, original gene expression data for ICI-treated solid cancers (*n* = 65; NSCLC, HNC, or melanoma) that were obtained with an identical methodology and previously published [9] were kindly provided by the authors for comparison (as the Prat cohort) with our original gene expression data for CUP. Gene expression was normalized on the basis of the data for the 40 housekeeping genes with the use of nSolver Analysis Software 4.0 and nCounter Advanced Analysis 2.0 (NanoString Technologies). Samples with abnormal normalized expression values (normalization factor of > 10 obtained with nSolver Analysis Software 4.0) were excluded, in accordance with the manufacturer’s instructions. A total of 135 RNA samples (72 from the CUP cohort, 63 from the Prat cohort) thus remained for further analysis. The cases of the Prat cohort were further divided into progressive disease (PD) and non-PD subsets on the basis of their best response to ICI treatment [9]. Of the 730 immune-related genes studied, 104 genes for which > 60% of samples showed an expression value below the minimum threshold were filtered out. Among the remaining 626 genes, 200 genes of biological interest were preselected for final analysis (Additional file 1: Table S1). The normalized gene expression data were log_2_-transformed before calculation of the Z score. Gene clustering was performed with the use of Cluster 3.0 software, and a heatmap was constructed with the use of Java TreeView.

### Statistical analysis

Fisher’s exact test and the Wilcoxon rank sum test were applied to compare categorical and continuous variables, respectively. Comparisons among more than two groups were performed with the Steel-Dwass test for multiple comparisons. Correlations were examined with the Spearman rank correlation test. The Benjamini-Hochberg method was used to calculate the false discovery rate (FDR) for multiple testing. Differences in OS curves constructed by the Kaplan-Meier method were assessed with the log-rank test, and univariable and multivariable Cox proportional hazard regression models were adopted to determine hazard ratios (HRs). Multivariable analysis of the unfavorable CUP subset was performed with adjustment for age (≥75 versus < 75 years), sex, Eastern Cooperative Oncology Group performance status (≥2 versus < 2), histology (undifferentiated versus otherwise), serum lactate dehydrogenase level (≥223 versus < 223 IU/L), serum albumin concentration (< 4.0 versus ≥4.0 mg/dL), peripheral blood lymphocyte count (< 1000 versus ≥1000/mL), metastatic pattern (multiple LNs only versus otherwise), brain metastases (present versus absent), and treatment (chemotherapy versus no chemotherapy). These factors were adopted as covariates because previous studies have suggested that they might affect prognosis of the unfavorable CUP subset [17–21]. The limit of the normal range served as the cutoff value for serum levels of lactate dehydrogenase and albumin as well as for the peripheral blood lymphocyte count. Missing data were not imputed. All *P* values were based on a two-sided hypothesis, with those of < 0.05 being considered statistically significant. Statistical analysis was performed with JMP software version 14.0.0 (SAS Institute), Stata/IC version 14.2 (StataCorp LP), or GraphPad Prism 7.0 (GraphPad Software).

## Results

### Survival outcome of the CUP cohort (full-analysis set)

**Fig. 2:**
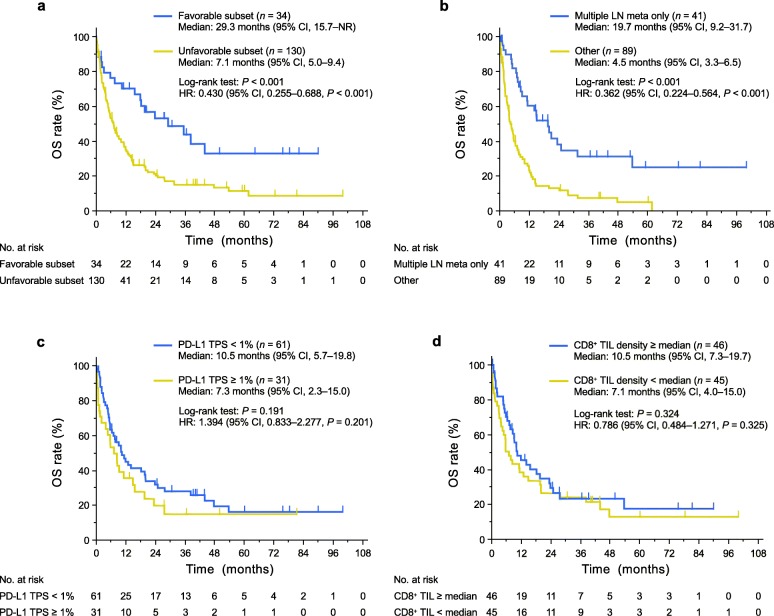
Kaplan-Meier curves for OS. **a** OS curves for the favorable and unfavorable subsets of patients with CUP in the full-analysis set. **b** OS curves for the unfavorable subset of CUP patients in the full-analysis set according to the prognostic metastatic (meta) pattern in which lesions are limited to multiple LNs. **c**, **d** OS curves for CUP patients in the biomarker-analysis set according either to the TPS for PD-L1 (**c**) or to CD8^+^ TIL density (**d**). One patient with only one cell block specimen available was excluded from the analysis of CD8^+^ TIL density because of the absence of tissue on the slide. Vertical lines on the curves denote censoring. NR, not reached

The characteristics of patients in the full-analysis set are shown in Table 1 and Additional file 2: Table S2. As expected, median OS was significantly longer in the favorable subset than in the unfavorable subset (HR of 0.430 with a 95% confidence interval [CI] of 0.255–0.688, *P* < 0.001) (Fig. 2a). However, the median OS was still only 29.3 months (95% CI, 15.7–not reached) and the estimated 5-year survival rate was only 32.8% even in the favorable subset, with the corresponding values for the unfavorable subset being 7.1 months (5.0–9.4) and 11.3%. Multivariable analysis revealed that a specific metastatic pattern in which the lesions are limited to multiple LNs was strongly prognostic for long-term survival in the unfavorable subset (Additional file 3: Table S3). Indeed, the median OS of patients with this metastatic pattern was significantly longer than that of those without it (19.7 versus 4.5 months, *P* < 0.001), with the 5-year survival rates being 24.8 and 4.8%, respectively (Fig. 2b).

**Table 1:** Characteristics of the study patients (*n* = 164)

Characteristic	No. of patients (%)^a^		*P* value^b^
	Full-analysis set (*n* = 164)	Biomarker-analysis set (*n* = 92)	
Median age (range), years	68 (35–95)	68 (35–95)	0.915
Sex			0.427
Male	94 (57)	58 (63)	
Female	70 (43)	34 (37)	
ECOG performance status			0.909
0–1	100 (61)	57 (62)	
2	34 (21)	17 (18)	
3–4	15 (9)	9 (10)	
Unknown (not recorded)	15 (9)	9 (10)	
Smoking history^c^			0.772
Current or former	93 (57)	57 (62)	
Never	49 (30)	27 (29)	
Unknown (not recorded)	22 (13)	8 (9)	
Favorable subset	34 (21)	20 (22)	0.874
Neuroendocrine carcinoma (NEC)	9 (5)	4 (4)	
Squamous carcinoma limited to cervical lymph nodes (HNC-like)	13 (8)	10 (11)	
Adenocarcinoma restricted to axillary lymph nodes in females (BC-like)	1 (1)	1 (1)	
Extragonadal germ cell tumor syndrome (GCT-like)	1 (1)	1 (1)	
Peritoneal carcinomatosis in females (PPC-like)	7 (4)	2 (2)	
Squamous carcinoma limited to inguinal lymph nodes (ACC-like)	1 (1)		
Single resectable metastatic carcinoma	2 (1)	2 (2)	
Unfavorable subset	130 (79)^d^	72 (78)^d^	
Histology			0.759
Squamous	33 (20)	21 (23)	
Adeno	76 (46)	42 (46)	
Undifferentiated	36 (22)	22 (24)	
Other	19 (12)^e^	7 (8)^f^	

*Abbreviations*: *ECOG* Eastern Cooperative Oncology Group

^a^Percentages may not add up to 100 because of rounding

^b^Fisher’s exact test

^c^Current smokers were defined as individuals who had smoked ≥100 cigarettes including at least one within the year prior to diagnosis; former smokers as those who had smoked ≥100 cigarettes but had quit > 1 year prior to diagnosis; and never-smokers as those who had smoked < 100 cigarettes

^d^A plausible primary site of origin was identified in one patient (primary differentiated thyroid carcinoma identified after second-line chemotherapy in a patient with multiple bone metastases)

^e^Adenosquamous, *n* = 3; neuroendocrine carcinoma, *n* = 9; not otherwise specified, *n* = 7

^f^Adenosquamous, *n* = 1; neuroendocrine carcinoma, *n* = 4; not otherwise specified, *n* = 2

### Immune profiling of CUP by IHC and irGEP (biomarker-analysis set)

We next evaluated the immune profile of patients in the biomarker-analysis set with the use of IHC and irGEP in order to explore the clinical potential of CUP for treatment with ICIs. The characteristics of patients in the biomarker-analysis set are shown in Table 1 and Additional file 2: Table S2. There was no significant difference in clinical features between the biomarker-analysis set and full-analysis set. The median OS for the biomarker-analysis set (Additional file 4: Figure S1a) was thus similar to that for the full-analysis set (Fig. 2a).

The proportion of individuals with a PD-L1 TPS of ≥1% was 34%, which is similar to values determined with the same monoclonal antibody (clone 28–8) for HNC and melanoma in clinical trials [22, 23]. The proportion of individuals with a PD-L1 CPS of ≥1% was 48%, which is similar to a value for gastric cancer [24]. PD-L1 positivity was not associated with survival outcome in our ICI-untreated CUP cohort (Fig. 2c and Additional file 4: Figure S1b). In addition, CD8^+^ TIL density was not associated with survival outcome (Fig. 2d). PD-L1 positivity and CD8^+^ TIL density did not differ significantly between the favorable and unfavorable subsets (PD-L1 TPS, *P* = 0.595; PD-L1 CPS, *P* = 0.317; CD8^+^ TIL density, *P* = 0.734), and these immune markers were also not associated with survival outcome even in the unfavorable subset (Additional file 5: Figure S2).

**Fig. 3:**
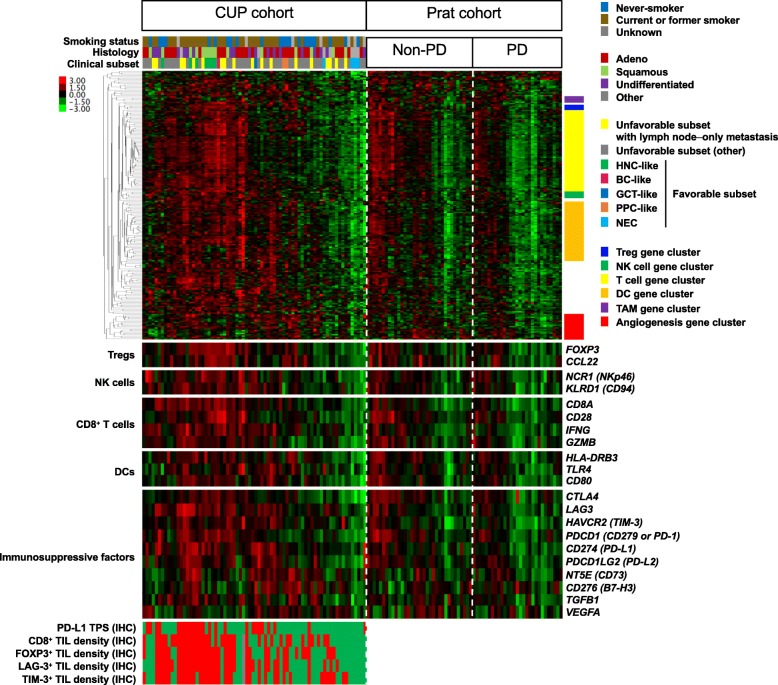
Heatmap of immune-related gene expression**.** CUP cohort (*n* = 72) was compared with ICI-treated solid cancers (Prat cohort, *n* = 63). The Prat cohort is divided into PD (*n* = 29) and non-PD (*n* = 34) subsets according to the best response to ICI treatment. Each colored square in the heatmap represents the Z score for the expression of one gene, with the highest expression shown in red, median in black, and lowest in green. Clinical characteristics are shown above the heatmap, gene clusters related to specific immune cell types on the right, and the expression of selected genes of interest below. Protein expression evaluated by IHC is shown at the bottom, with red and green boxes representing ≥1 and < 1% for PD-L1 TPS, and with red, green, and gray boxes representing ≥median, <median, and not examined for TIL density (because only a cell block specimen with no tissue on the slide was available), respectively. Treg, regulatory T cell; TAM, tumor-associated macrophage

**Fig. 4:**
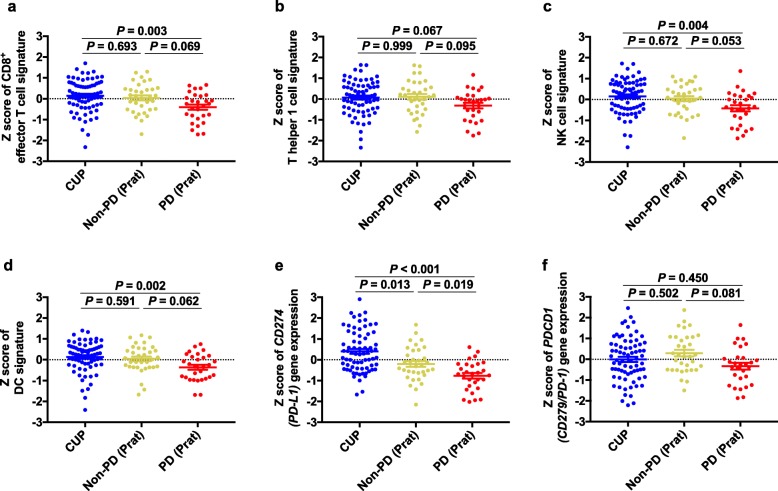
Dot plots for antitumor immune gene expression signatures. CUP cohort (*n* = 72) was compared with the Prat cohort of ICI-treated solid cancers (*n* = 63). **a**–**d** Gene signatures for CD8^+^ effector T cells, T helper 1 cells, NK cells, and DCs, respectively. **e, f** Expression of *CD274 (PD-L1)* and *PDCD1 (CD279 or PD-1)* genes, respectively. The Prat cohort is divided into PD (*n* = 29) and non-PD (*n* = 34) subsets according to the best response to ICI treatment. Each dot represents one patient. The mean and standard error of the mean values are shown for each plot. *P* values were determined with the Steel-Dwass test for multiple comparisons

We performed irGEP to evaluate the immune profile of CUP in more detail (Fig. 3). The characteristics of the 72 patients in the biomarker-analysis set with irGEP data were similar to those of the patients (*n* = 164) in the full-analysis set and those of the entire group of patients (*n* = 92) in the biomarker-analysis set (Table 1 and Additional file 6: Table S4). Gene expression values for PD-L1 (*P* < 0.001) and TIL markers (*r* = 0.49–0.74, *P* < 0.001) were well correlated with the IHC results (Additional file 7: Figure S3). Gene expression data for ICI-treated solid cancers (Prat cohort) were also analyzed as a comparator group. Of note, antitumor immune-related gene expression (Additional file 8: Table S5) [9, 10, 25–28]—including that related to T cells, natural killer (NK) cells, or dendritic cells (DCs)—was similar for the CUP cohort and the non-PD subset of the Prat cohort, whereas that for the PD subset of the Prat cohort was significantly lower or tended to be lower (Fig. 4). In addition, inhibitory immune checkpoint molecules responsible for escape from antitumor immunity were expressed in CUP as well as in the Prat cohort. These immune cell–related gene expression signatures predictive of response to ICI treatment did not differ significantly between the favorable and unfavorable subsets of CUP (Additional file 9: Figure S4a). Among the favorable subset of CUP, HNC-like tumors showed relatively high levels of expression for these gene signatures whereas NEC showed low levels. In addition, the prognostic metastatic pattern of the unfavorable subset was not associated with these antitumor immune signatures (Additional file 9: Figure S4b). Furthermore, neither smoking status nor histology was associated with the CD8^+^ effector T cell or T helper 1 cell gene signatures (Additional file 9: Figure S4c, d).

**Fig. 5:**
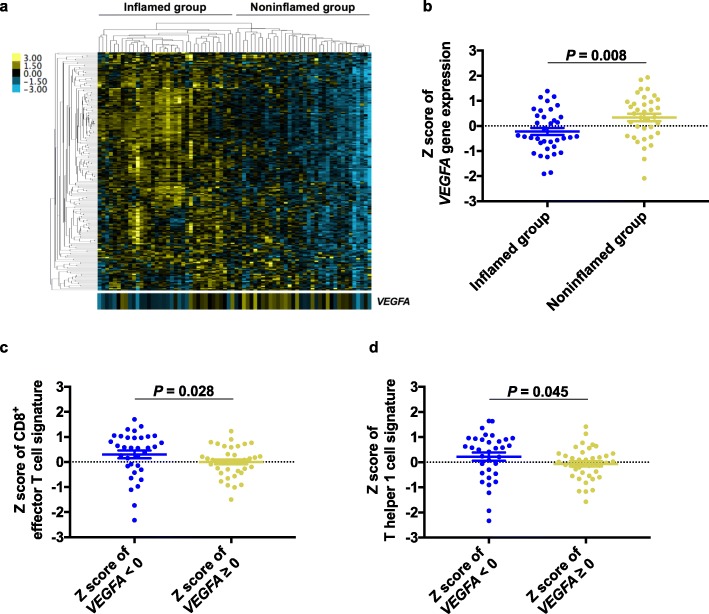
Inverse association of *VEGFA* expression with expression of antitumor immune gene signatures. **a** Heatmap of immune-related gene expression for inflamed and noninflamed subsets of the CUP cohort (*n* = 72). Each colored square in the heatmap represents the Z score for the expression of one gene, with the highest expression shown in yellow, median in black, and lowest in blue. Expression of *VEGFA* is shown below. **b**–**d** Dot plots of *VEGFA* expression for the inflamed group (*n* = 36) and the noninflamed group (*n* = 36) of the CUP cohort (**b**) as well as of CD8^+^ effector T cell (**c**) and T helper 1 cell (**d**) gene expression signatures for the CUP cohort (*n* = 72) according to *VEGFA* expression (Z score < 0, *n* = 34; Z score ≥ 0, *n* = 38). The FDR in (**b**) was 0.010. The mean and SEM values are shown, and the *P* values were determined with the Wilcoxon rank sum test

To explore genes whose expression was negatively associated with antitumor immunity in CUP, we divided the patients with CUP into inflamed (enriched for immune-related gene expression) and noninflamed groups on the basis of their gene clustering pattern and then compared the expression levels of each of the 200 immune-related genes between the two groups (Fig. 5a). The vascular endothelial growth factor–A *(VEGFA)* gene was the only gene that was expressed at a significantly higher level in the noninflamed group (*P* = 0.008, FDR = 0.010) (Fig. 5b). *VEGFA* expression was associated with low levels of expression for the gene signatures of both CD8^+^ effector T cells and T helper 1 cells (Fig. 5c, d).

### Clinical benefit of nivolumab treatment in a case of CUP in the unfavorable subset

Only one case received ICI treatment during the study period, and this patient responded well to the immunotherapy (Fig. 1). The patient was a 78-year-old female never-smoker who was diagnosed with CUP of the unfavorable subset after thorough clinical examination based on clinical practice guidelines at age 76. The lesions were distributed in multiple LNs (multiple LNs metastasis–only pattern), which could not be resected and were irradiated with curative intent (Additional file 10: Figure S5a). A surgical biopsy of the left supraclavicular lesion revealed low-differentiated squamous cell carcinoma with prominent PD-L1 expression on immune cells and CD8^+^ lymphocyte infiltration in the tumor, but no PD-L1 expression on tumor cells (Additional file 10: Figure S5b). The patient received platinum-doublet chemotherapy as a first-line treatment, which resulted in disease progression after ~ 1 year. The occult primary tumor was clinically predicted to be HNC or NSCLC on the basis of histology and the distribution pattern of the lesions [1]. The patient was therefore treated with nivolumab as second-line therapy, which resulted in tumor regression and durable disease control (Additional file 10: Figure S5c). At the time of writing, she remains without disease progression after 7 months of the treatment.

## Discussion

The clinical review of our study cohort revealed that the survival outcome of CUP remains unsatisfactory. However, our IHC and irGEP data showed that CUP has immune characteristics suitable for treatment with ICIs that were similar to those of ICI-responsive solid cancers. In addition, the clinical course of a CUP patient who experienced a response to nivolumab treatment supported this notion. As far as we are aware, our study is the first to investigate the immune profile of CUP with direct analyses of immune phenotype by IHC and irGEP.

Only a few previous studies have investigated the immune profile of CUP [8, 29]. With the application of IHC, these studies identified a subset of CUP patients with PD-L1 expression on their tumor cells and programmed cell death–1 (PD-1) expression on TILs, consistent with our findings. The tumor mutation burden of CUP was also shown to be similar to that of ICI-responsive malignancies such as NSCLC and bladder cancer, whereas mismatch repair (MMR) deficiency was infrequently observed [29]. Although PD-L1 expression in tumors and tumor mutation burden are widely accepted as biomarkers for PD-1/PD-L1 inhibitor treatment across many cancer types, more detailed and direct immunologic analyses of TILs including irGEP have been proposed to provide additional biomarkers [6, 9, 10, 14, 24, 30, 31]. Our comprehensive analyses by IHC and irGEP provide further support for the notion that patients with CUP can receive clinical benefit from ICI treatment. Previous studies of metastatic NSCLC found that PD-L1 expression in tumors and immune signatures were not associated with the efficacy of non-ICI treatment [30]. In addition, an immune infiltration phenotype based on irGEP was not associated with survival in patients with MMR-deficient cancer in the pre-ICI era [32]. These observations indicate that immune activity contributes to the survival outcome of patients with metastatic cancer only if they are treated with ICIs. Indeed, PD-L1 expression and CD8^+^ TIL density were not associated with survival outcome in our CUP cohort treated with non-ICI therapy. ICI treatment might therefore be expected to improve the survival outcome of CUP patients compared with that currently achieved with conventional therapies.

We also explored whether various subsets of CUP patients might be more suitable for ICI treatment than others. However, none of the clinical characteristics examined was associated with immune activity in CUP. Both favorable and unfavorable subsets of CUP patients thus showed equal potential to receive benefit from ICI treatment. Furthermore, prognostic metastatic pattern among the unfavorable subset, smoking status, and histology were not associated with an ICI-responsive immune profile. These findings again emphasize that the survival outcome of CUP patients is not linked to immune activity if they are not treated with ICIs, and they further show that all CUP patients have a similar potential to receive benefit from ICI treatment. Among the favorable subset of CUP patients, those with HNC-like lesions were more likely to have a desirable immune phenotype, whereas those with NEC were less likely. These findings remain inconclusive because of the small number of cases, but they may support preferential treatment of patients with HNC-like lesions with ICIs.

ICI combination therapy is currently under development for various types of cancer in order to overcome an insufficient treatment outcome with ICI monotherapy [6, 33, 34]. The components of such combination therapy include novel ICIs, antiangiogenesis agents, and cytotoxic chemotherapeutic drugs. Our comprehensive irGEP indicated that tumor tissue in CUP patients expresses the targets of such novel ICIs including LAG-3, TIM-3, CD73, B7-H3, and transforming growth factor–β (TGF-β) as well as those of conventional ICIs including PD-L1, PD-L2, PD-1, and cytotoxic T lymphocyte antigen–4 (CTLA-4). Our preliminary analysis further indicated that *VEGFA* expression was associated with suppression of antitumor immunity, suggesting that VEGF-A blockade may enhance ICI efficacy in CUP patients.

There are several limitations to our study. The study was thus retrospective in nature and the number of patients was relatively small, making it difficult to overcome clinical heterogeneity of CUP. Next-generation sequencing assays as well as molecular gene expression profiling assays such as 92-gene reverse transcriptase–polymerase chain reaction–based cancer classification assay [3], were not approved for CUP patients in Japan during the study period, which thus precluded the collection of data for molecular prediction of the primary site. In addition, none of the patients was subjected to postmortem examination. The association of primary site predicted or determined by such modalities with immune profile therefore needs to be clarified in future studies. Nevertheless, our comprehensive evaluation with IHC and irGEP, which included comparison of our CUP cohort with an ICI-treated cohort of solid cancers, yielded consistent findings with regard to the immune profile of CUP, indicative of the potential of CUP to be treated with ICIs. Prospective clinical trials to confirm the efficacy of ICIs in CUP patients are thus warranted. Indeed, several trials evaluating the efficacy of ICIs in CUP patients are currently in progress, including phase II trials of pembrolizumab (NCT03391973 and NCT03752333) as well as our phase II trial of nivolumab (NivoCUP, UMIN-CTR ID UMIN000030649).

## Conclusions

Our comprehensive immunologic analyses have revealed that the immune profile of CUP is similar to that of ICI-responsive malignancies, and they thus suggest that CUP patients will receive clinical benefit from ICI treatment. Our study therefore provides a rationale for prospective clinical trials of immunotherapy for CUP.

## Additional files

Additional file 1: **Table S1.** The 200 predetermined genes of interest. (DOCX 23 kb)

Additional file 2: **Table S2.** Detailed characteristics of the unfavorable subset of CUP patients. (DOCX 26 kb)

Additional file 3: **Table S3.** Cox proportional hazard regression analysis of the effect of clinical factors on OS in the unfavorable subset of CUP patients (*n* = 130). (DOCX 22 kb)

Additional file 4: **Figure S1.** Kaplan-Meier curves for OS of patients in the biomarker-analysis set. (DOCX 175 kb)

Additional file 5: **Figure S2.** Kaplan-Meier curves for OS of patients in the unfavorable subset of the biomarker-analysis set. (DOCX 244 kb)

Additional file 6: **Table S4.** Detailed characteristics of the patients analyzed by irGEP. (DOCX 16 kb)

Additional file 7: **Figure S3.** Comparison of gene expression scores with IHC scores for CUP (*n* = 72). (DOCX 240 kb)

Additional file 8: **Table S5.** Predetermined genes for each immune cell signature. (DOCX 23 kb)

Additional file 9: **Figure S4.** Antitumor immune gene expression signatures for CUP patients according to clinical characteristics. (DOCX 384 kb)

Additional file 10: **Figure S5.** A case of CUP of the unfavorable subset treated with nivolumab. (DOCX 1800 kb)

## Data Availability

The datasets used and/or analyzed during the current study are available from the corresponding author on reasonable request.

## Abbreviations

ACC: Anal canal carcinoma
BC: Breast cancer
CI: Confidence interval
CPS: Combined positive score
CTLA-4: Cytotoxic T lymphocyte antigen–4
CUP: Cancer of unknown primary site
DC: Dendritic cell
FDG-PET/CT: ^18^F-fluoro-2-deoxy-d-glucose–computed tomography scans
FDR: False discovery rate
FFPE: Formalin-fixed paraffin-embedded
FOXP3: Forkhead box P3
GCT: Germ cell tumor
HE: Hematoxylin-eosin
HNC: Head and neck cancer
HR: Hazard ratio
ICI: Immune checkpoint inhibitor
IHC: Immunohistochemistry
irGEP: Immune-related gene expression profiling
LAG-3: Lymphocyte activation gene-3
LN: Lymph node
MMR: Mismatch repair
NEC: Neuroendocrine carcinoma
NK: Natural killer
NSCLC: Non–small cell lung cancer
OS: Overall survival
PD: Progressive disease
PD-1: Programmed cell death–1
PD-L1: Programmed cell death–ligand 1
PPC: Primary peritoneal cancer
TGF-β: Transforming growth factor–β
TIL: Tumor-infiltrating lymphocyte
TIM-3: T cell immunoglobulin and mucin domain-3
TPS: Tumor proportion score
VEGFA: Vascular endothelial growth factor A
