# Supplementary material for: Clinical and immune profiling for cancer of unknown primary site
Source: J Immunother Cancer. 2019 Sep 13;7:251. doi: 10.1186/s40425-019-0720-z (PMC6743146; doi:10.1186/s40425-019-0720-z)
Supplement: Supplementary file 1 — Table S1. The 200 predetermined genes of interest. (DOCX 23 kb) [file 40425_2019_720_MOESM1_ESM.docx]

| **Table S1. The 200 predetermined genes of interest** | | | | |
| --- | --- | --- | --- | --- |
| ADORA2A | CD6 | GZMH | ITGAL (CD11a/LFA-1) | SELE (selectinE) |
| AXL | CD68 | GZMK | ITGAM (CD11b) | SELPLG |
| BTLA | CD7 | GZMM | ITGAX (CD11c) | SLAMF6 |
| CCL17 | CD74 | HAVCR2 (TIM-3) | ITK | SLAMF7 |
| CCL19 | CD80 | HLA-DMA | JAK1 | SPN (CD43) |
| CCL2 | CD83 | HLA-DMB | JAK2 | STAT1 |
| CCL22 | CD84 | HLA-DOB | KLRB1 (NK1.1) | STAT4 |
| CCL3 | CD86 | HLA-DPA1 | KLRC1 (NKG2A) | TBX21 (T-bet) |
| CCL3L1 | CD8A | HLA-DPB1 | KLRC2 (NKG2C) | TCF7 |
| CCL4 | CD8B | HLA-DRA | KLRD1 (CD94) | TGFB1 |
| CCL5 | CD96 | HLA-DRB3 | KLRF1 (NKp80) | TICAM1 |
| CCL8 | CDH5 (Vecadherin) | ICAM3 | KLRG1 | TICAM2 |
| CCR1 | CLEC7A | ICOS | KLRK1 (NKG2D) | TIGIT |
| CCR2 | CMKLR1 | ICOSLG | LAG3 | TLR1 |
| CCR4 | CSF1R | IDO1 | LCK | TLR10 |
| CCR5 | CSF2RB | IFNG | LGALS3 (Galectin3) | TLR2 |
| CCR6 | CSF3R | IL10 | LILRB1 | TLR3 |
| CCR7 | CTLA4 | IL12A | LRP1 (CD91) | TLR4 |
| CD14 | CX3CL1 | IL12RB1 | LTA | TLR5 |
| CD163 | CX3CR1 | IL12RB2 | LTB | TLR6 |
| CD180 | CXCL1 | IL15RA | LY86 | TLR7 |
| CD209 | CXCL10 | IL16 | LY96 | TLR8 |
| CD244 (2B4) | CXCL11 | IL18 | LYN | TLR9 |
| CD247 (CD3Z) | CXCL12 | IL18R1 | MARCO | TNF |
| CD27 | CXCL16 | IL18RAP | MRC1 (CD206) | TNFRSF18 (GITR) |
| CD274 (PD-L1) | CXCL9 | IL1A | MSR1 (CD204) | TNFRSF4 (CD134/OX40) |
| CD276 (B7-H3) | CXCR1 | IL1B | NCR1 (NKp46) | TNFRSF8 |
| CD28 | CXCR2 | IL21R | NOD2 | TNFRSF9 (4-1BB) |
| CD33 | CXCR3 | IL2RA (CD25) | NRP1 | TNFSF10 (TRAIL) |
| CD34 | CXCR4 | IL2RB (CD122) | NT5E (CD73) | TNFSF11 (RANKL) |
| CD3D | CYBB (NOX2) | IL2RG (CD132) | PDCD1 (CD279/PD-1) | TNFSF12 |
| CD3E | ENTPD1 (CD39) | IL7 | PDCD1LG2 (PD-L2) | TNFSF4 (OX40L) |
| CD3G | EOMES | IL7R | PDGFC | TNFSF8 |
| CD4 | FCGR1A | IL8 | PDGFRB | TXK |
| CD40 | FCGR2A | IRF1 | PECAM1 (CD31) | VCAM1 |
| CD40LG | FCGR2B | IRF4 | PRF1 | VEGFA |
| CD44 | FCGR3A | IRF8 | PSMB10 | VEGFC |
| CD47 | FOXP3 | ITGA4 | PTPRC (CD45) | XCL2 |
| CD5 | GZMA | ITGA5 (CD49e) | REL | XCR1 |
| CD58 | GZMB | ITGAE (CD103) | RUNX1 | ZAP70 |
