# Supplementary material for: Clinical and immune profiling for cancer of unknown primary site
Source: J Immunother Cancer. 2019 Sep 13;7:251. doi: 10.1186/s40425-019-0720-z (PMC6743146; doi:10.1186/s40425-019-0720-z)
Supplement: Supplementary file 2 — Table S2. Detailed characteristics of the unfavorable subset of CUP patients. (DOCX 26 kb) [file 40425_2019_720_MOESM2_ESM.docx]

| **Table S2. Detailed characteristics of the unfavorable subset of CUP patients** | | | |
| --- | --- | --- | --- |
| **Characteristic** | **No. of patients (%)^a^** | | ***P* value^b^** |
|  | **Full-analysis set**  **(*n* = 130)** | **Biomarker-analysis set**  **(*n* = 72)** |  |
| Median age (range), years | 68 (37–95) | 68 (37–95) | 0.871 |
| Sex |  |  | 0.456 |
| Male | 73 (56) | 45 (63) |  |
| Female | 57 (44) | 27 (38) |  |
| ECOG performance status |  |  | 0.942 |
| 0–1 | 80 (62) | 46 (64) |  |
| 2 | 25 (19) | 12 (17) |  |
| 3–4 | 13 (10) | 7 (10) |  |
| Unknown (not recorded) | 12 (9) | 7 (10) |  |
| Smoking history^c^ |  |  | 0.621 |
| Current or former | 72 (55) | 45 (63) |  |
| Never | 39 (30) | 20 (28) |  |
| Unknown (not recorded) | 19 (15) | 7 (10) |  |
| Metastatic lesions |  |  |  |
| CNS | 8 (6) | 3 (4) | 0.750 |
| Multiple lymph nodes only | 41 (32) | 24 (33) | 0.875 |
| Histology |  |  | 0.853 |
| Squamous | 17 (13) | 9 (13) |  |
| Adeno | 68 (52) | 39 (54) |  |
| Undifferentiated | 35 (27) | 21 (29) |  |
| Other | 10 (8)^d^ | 3 (4)^e^ |  |
| Serum albumin |  |  | 1.000 |
| Normoalbuminemia (≥4.0 mg/dL) | 36 (28) | 20 (28) |  |
| Hypoalbuminemia (<4.0 mg/dL) | 92 (71) | 52 (72) |  |
| Not examined | 2 (2) |  |  |
| Serum LDH |  |  | 1.000 |
| Not elevated (<223 IU/L) | 58 (45) | 32 (44) |  |
| Elevated (≥223 IU/L) | 71 (55) | 40 (56) |  |
| Not examined | 1 (1) |  |  |
| Peripheral blood lymphocyte count |  |  | 0.728 |
| ≥1000/mL | 100 (77) | 54 (75) |  |
| <1000/mL | 28 (22) | 17 (24) |  |
| Not examined | 2 (2) | 1 (1) |  |
| Treatment |  |  | 0.871 |
| Chemotherapy | 94 (72) | 51 (71) |  |
| Best supportive care only | 36 (28) | 21 (29) |  |
| Chemotherapy regimen |  |  | 0.786 |
| Platinum-based doublet or triplet | 74 (79) | 38 (75) |  |
| Cytotoxic monotherapy | 12 (13) | 8 (16) |  |
| Molecularly-targeted therapy | 8 (9) | 5 (10) |  |
| Abbreviations: CUP, cancer of unknown primary site; ECOG, Eastern Cooperative Oncology Group; CNS, central nervous system; LDH, lactate dehydrogenase. | | | |
| ^a^Percentages may not add up to 100 because of rounding. | | | |
| ^b^Fisher’s exact test. | | | |
| ^c^Current smokers were defined as individuals who had smoked ≥100 cigarettes including at least one within the year prior to diagnosis; former smokers as those who had smoked ≥100 cigarettes but had quit >1 year prior to diagnosis; and never-smokers as those who had smoked <100 cigarettes. | | | |
| ^d^Adenosquamous, *n* = 3; not otherwise specified, *n* = 7. | | | |
| ^e^Adenosquamous, *n* = 1; not otherwise specified, *n* = 2. | | | |
