# Supplementary material for: Clinical and immune profiling for cancer of unknown primary site
Source: J Immunother Cancer. 2019 Sep 13;7:251. doi: 10.1186/s40425-019-0720-z (PMC6743146; doi:10.1186/s40425-019-0720-z)
Supplement: Supplementary file 3 — Table S3. Cox proportional hazard regression analysis of the effect of clinical factors on OS in the unfavorable subset of CUP patients (n = 130). (DOCX 22 kb) [file 40425_2019_720_MOESM3_ESM.docx]

| **Table S3. Cox proportional hazard regression analysis of the effect of clinical factors on OS in the unfavorable subset of CUP patients (*n* = 130)** | | | | | | | |
| --- | --- | --- | --- | --- | --- | --- | --- |
| **Factor** | **Univariable** | | | **Multivariable** | | |  |
|  | **Hazard ratio** | **95% CI** | ***P* value** | **Hazard ratio** | **95% CI** | ***P* value** |  |
| Age ≥75 years | 1.213 | 0.729–1.928 | 0.443 | 0.951 | 0.497–1.771 | 0.877 |  |
| Male sex | 1.431 | 0.966–2.141 | 0.074 | 1.708 | 1.070–2.765 | 0.025 |  |
| ECOG PS ≥2 | 2.660 | 1.672–4.147 | <0.001 | 1.344 | 0.773–2.289 | 0.290 |  |
| Undifferentiated carcinoma | 1.288 | 0.836–1.941 | 0.246 | 1.362 | 0.837–2.176 | 0.209 |  |
| Elevated serum LDH | 1.589 | 1.068–2.386 | 0.022 | 1.642 | 1.042–2.622 | 0.033 |  |
| Hypoalbuminemia | 2.223 | 1.438–3.530 | <0.001 | 1.563 | 0.952–2.639 | 0.078 |  |
| Peripheral blood lymphocyte count <1000/mL | 1.399 | 0.848–2.214 | 0.182 | 1.238 | 0.686–2.177 | 0.470 |  |
| Metastasis limited to multiple lymph nodes | 0.362 | 0.224–0.564 | <0.001 | 0.365 | 0.202–0.637 | <0.001 |  |
| CNS metastasis | 2.111 | 0.886–4.243 | 0.087 | 0.909 | 0.337–2.066 | 0.833 |  |
| Chemotherapy | 0.446 | 0.283–0.728 | 0.002 | 0.363 | 0.185–0.734 | 0.005 |  |
| Abbreviations: OS, overall survival; CUP, cancer of unknown primary site; CI, confidence interval; ECOG PS, Eastern Cooperative Oncology Group performance status; LDH, lactate dehydrogenase; CNS, central nervous system. | | | | | | | |
