# Supplementary material for: Clinical and immune profiling for cancer of unknown primary site
Source: J Immunother Cancer. 2019 Sep 13;7:251. doi: 10.1186/s40425-019-0720-z (PMC6743146; doi:10.1186/s40425-019-0720-z)
Supplement: Supplementary file 4 — Figure S1. Kaplan-Meier curves for OS of patients in the biomarker-analysis set. (DOCX 175 kb) [file 40425_2019_720_MOESM4_ESM.docx]

**Figure S1**

**Figure S1. Kaplan-Meier curves for OS of patients in the biomarker-analysis set.**

(**a**) Overall survival **(**OS) of patients in the favorable or unfavorable subsets. (**b**) OS of patients according to programmed cell death–ligand 1 (PD-L1) combined positive score (CPS). Vertical lines on the curves denote censoring. CI, confidence interval; NR, not reached; HR, hazard ratio.
