# Supplementary material for: Clinical and immune profiling for cancer of unknown primary site
Source: J Immunother Cancer. 2019 Sep 13;7:251. doi: 10.1186/s40425-019-0720-z (PMC6743146; doi:10.1186/s40425-019-0720-z)
Supplement: Supplementary file 5 — Figure S2. Kaplan-Meier curves for OS of patients in the unfavorable subset of the biomarker-analysis set. (DOCX 244 kb) [file 40425_2019_720_MOESM5_ESM.docx]

**Figure S2**

**Figure S2. Kaplan-Meier curves for OS of patients in the unfavorable subset of the biomarker-analysis set.**

Overall survival (OS) of patients was examined according to programmed cell death–ligand 1 (PD-L1) tumor proportion score (TPS) (**a**), PD-L1 combined positive score (CPS) (**b**) or CD8^+^ tumor-infiltrating lymphocyte (TIL) density (**c**). Vertical lines on the curves denote censoring. CI, confidence interval; HR, hazard ratio.
