# Supplementary material for: Clinical and immune profiling for cancer of unknown primary site
Source: J Immunother Cancer. 2019 Sep 13;7:251. doi: 10.1186/s40425-019-0720-z (PMC6743146; doi:10.1186/s40425-019-0720-z)
Supplement: Supplementary file 6 — Table S4. Detailed characteristics of the patients analyzed by irGEP. (DOCX 16 kb) [file 40425_2019_720_MOESM6_ESM.docx]

| **Table S4. Detailed characteristics of the patients analyzed by irGEP (*n* = 72)** | |
| --- | --- |
| **Characteristic** | **No. of patients (%)^a^** |
| Median age (range), years | 67 (35–95) |
| Sex |  |
| Male | 43 (60) |
| Female | 29 (40) |
| ECOG performance status |  |
| 0–1 | 42 (58) |
| 2 | 15 (21) |
| 3–4 | 8 (11) |
| Unknown (not recorded) | 7 (10) |
| Smoking history^b^ |  |
| Current or former | 43 (60) |
| Never | 22 (31) |
| Unknown (not recorded) | 7 (10) |
| Favorable subset | 15 (21) |
| Neuroendocrine carcinoma (NEC) | 4 (6) |
| Squamous carcinoma limited to cervical lymph nodes (HNC-like) | 7 (10) |
| Adenocarcinoma restricted to axillary lymph nodes in females (BC-like) | 1 (1) |
| Extragonadal germ cell tumor syndrome (GCT-like) | 1 (1) |
| Peritoneal carcinomatosis in females (PPC-like) | 2 (3) |
| Squamous carcinoma limited to inguinal lymph nodes (ACC-like) | 0 (0) |
| Single resectable metastatic carcinoma | 0 (0) |
| Unfavorable subset | 57 (79)**^c^** |
| Multiple lymph nodes only metastasis | 19 (33) |
| Histology |  |
| Squamous | 15 (21) |
| Adeno | 32 (44) |
| Undifferentiated | 18 (25) |
| Other | 7 (10)^d^ |
| Abbreviations: irGEP, immune-related gene expression profiling; ECOG, Eastern Cooperative Oncology Group. | |
| ^a^Percentages may not add up to 100 because of rounding. | |
| ^b^Current smokers were defined as individuals who had smoked ≥100 cigarettes including at least one within the year prior to diagnosis; former smokers as those who had smoked ≥100 cigarettes but had quit >1 year prior to diagnosis; and never-smokers as those who had smoked <100 cigarettes. | |
| ^c^A plausible primary site of origin was identified in one patient (primary differentiated thyroid carcinoma was identified after second-line chemotherapy in a patient with multiple bone metastases). | |
| ^d^Adenosquamous, *n* = 1; neuroendocrine carcinoma, *n* = 4; not otherwise specified, *n* = 2. | |
