# Supplementary material for: Clinical and immune profiling for cancer of unknown primary site
Source: J Immunother Cancer. 2019 Sep 13;7:251. doi: 10.1186/s40425-019-0720-z (PMC6743146; doi:10.1186/s40425-019-0720-z)
Supplement: Supplementary file 7 — Figure S3. Comparison of gene expression scores with IHC scores for CUP (n = 72). (DOCX 240 kb) [file 40425_2019_720_MOESM7_ESM.docx]

**Figure S3**

**Figure S3. Comparison of gene expression scores with IHC scores for CUP (*n* = 72).**

(**a, b**) Dot plot of *CD274 (PD-L1)* gene expression score according to the PD-L1 tumor proportion score (TPS) (<1%, *n* = 47; ≥1%, *n* = 25) (**a**) or PD-L1 combined positive score (CPS) (<1%, *n* = 35; ≥1%, *n* = 37) (**b**) determined by immunohistochemistry (IHC). The mean and standard error of the mean values are shown, and the *P* values were determined with the Wilcoxon rank sum test. (**c, d, e, f**) Scatter plots of CD8^+^ (**c**), forkhead box P3 (FOXP3)^+^ (**d**), lymphocyte activation gene–3 (LAG-3)^+^ (**e**), or T cell immunoglobulin and mucin domain–3 (TIM-3)^+^ (**f**) tumor-infiltrating lymphocyte (TIL) density determined by IHC versus the corresponding gene expression score. The Spearman rank correlation coefficient (*r*) and *P* values are shown. CUP, cancer of unknown primary site.
