# Supplementary material for: Clinical and immune profiling for cancer of unknown primary site
Source: J Immunother Cancer. 2019 Sep 13;7:251. doi: 10.1186/s40425-019-0720-z (PMC6743146; doi:10.1186/s40425-019-0720-z)
Supplement: Supplementary file 8 — Table S5. Predetermined genes for each immune cell signature. (DOCX 23 kb) [file 40425_2019_720_MOESM8_ESM.docx]

| **Table S5. Predetermined genes for each immune cell signature** | | | |
| --- | --- | --- | --- |
| **CD8^+^ effector T cells** | **T helper 1 cells** | **Natural killer cells** | **Dendritic cells** |
| CD247 (CD3Z) | BTLA | CD244 (2B4) | CD40 |
| CD27 | CD247 (CD3Z) | GZMA | CD58 |
| CD28 | CD3D | GZMB | CD74 |
| CD3D | CD3E | GZMH | CD80 |
| CD3E | CD3G | GZMK | CD83 |
| CD3G | CD4 | GZMM | CD86 |
| CD44 | CD40LG | KLRB1 (NK1.1) | CLEC7A |
| CD5 | CD44 | KLRC1 (NKG2A) | CXCL10 |
| CD7 | IFNG | KLRC2 (NKG2C) | CXCL11 |
| CD8A | IL12RB1 | KLRD1 (CD94) | CXCL16 |
| CD8B | IL12RB2 | KLRF1 (NKp80) | CXCL9 |
| CX3CR1 | IL2RB (CD122) | KLRK1 (NKG2D) | HLA-DMA |
| CXCR3 | IL2RG (CD132) | NCR1 (NKp46) | HLA-DMB |
| EOMES | LCK |  | HLA-DOB |
| GZMA | LTA |  | HLA-DPA1 |
| GZMB | STAT4 |  | HLA-DPB1 |
| GZMH | TBX21 (T-bet) |  | HLA-DRA |
| GZMK | TXK |  | HLA-DRB3 |
| GZMM | ZAP70 |  | IRF4 |
| ICOS |  |  | ITGAM (CD11b) |
| IFNG |  |  | ITGAX (CD11c) |
| IL12RB1 |  |  | LY86 |
| IL12RB2 |  |  | TLR1 |
| IL15RA |  |  | TLR10 |
| IL2RB (CD122) |  |  | TLR2 |
| IL2RG (CD132) |  |  | TLR3 |
| IL7R |  |  | TLR4 |
| ITGA4 (VLA-4) |  |  | TLR5 |
| ITGAE (CD103) |  |  | TLR6 |
| ITGAL (CD11a/LFA-1) |  |  | TLR7 |
| KLRG1 |  |  | TLR8 |
| LCK |  |  | TLR9 |
| PRF1 |  |  |  |
| TBX21 (T-bet) |  |  |  |
| TNF |  |  |  |
| TNFRSF18 (GITR) |  |  |  |
| XCR1 |  |  |  |
| ZAP70 |  |  |  |
