# Supplementary material for: Clinical and immune profiling for cancer of unknown primary site
Source: J Immunother Cancer. 2019 Sep 13;7:251. doi: 10.1186/s40425-019-0720-z (PMC6743146; doi:10.1186/s40425-019-0720-z)
Supplement: Supplementary file 9 — Figure S4. Antitumor immune gene expression signatures for CUP patients according to clinical characteristics. (DOCX 384 kb) [file 40425_2019_720_MOESM9_ESM.docx]

**Figure S4**

**Figure S4. Antitumor immune gene expression signatures for CUP patients according to clinical characteristics.**

(**a**) Unfavorable (*n* = 57) and favorable (*n* = 15) subsets of patients. The dot plots of the favorable subset are further color-coded on the basis of clinical presentation. (**b**) Unfavorable subset of patients according to metastasis pattern (with [*n* = 18] or without [other, *n* = 39] the good prognostic metastatic pattern of multiple lymph node [LN] metastases [meta] only). (**c**) Seven cases were excluded because of missing data for smoking history (current or former smokers [*n* = 43] and never-smokers [*n* = 22]). (**d**) Adenocarcinoma (Adeno, *n* = 31), squamous carcinoma (*n* = 15), and undifferentiated carcinoma (*n* = 19). Cases of adenosquamous carcinoma (*n* = 1), neuroendocrine carcinoma (*n* = 4), or not otherwise specified histology (*n* = 2) were excluded from the analysis because of their small numbers. The mean and standard error of the mean values are shown for all plots, and the *P* values were determined with the Wilcoxon rank sum test (**a**–**c**) or the Steel-Dwass test (**d**). CUP, cancer of unknown primary site; NK, natural killer; DC, dendritic cell; HNC, head and neck cancer; BC, breast cancer; GCT, germ cell tumor; PPC, primary peritoneal cancer; NEC, neuroendocrine carcinoma.
