# Supplementary material for: Clinical and immune profiling for cancer of unknown primary site
Source: J Immunother Cancer. 2019 Sep 13;7:251. doi: 10.1186/s40425-019-0720-z (PMC6743146; doi:10.1186/s40425-019-0720-z)
Supplement: Supplementary file 10 — Figure S5. A case of CUP of the unfavorable subset treated with nivolumab. (DOCX 1800 kb) [file 40425_2019_720_MOESM10_ESM.docx]

**Figure S5**

**Figure S5. A case of CUP of the unfavorable subset treated with nivolumab.**

(**a**) Positron emission tomography with ^18^F-fluoro-2-deoxy-d-glucose scan showing metastases in multiple lymph nodes without an obvious primary site. (**b**) hematoxylin-eosin (HE) staining as well as immunohistochemical analysis of CD8 and programmed cell death–ligand 1 (PD-L1) for tumor biopsy tissue. CD8^+^ tumor-infiltrating lymphocyte (TIL) density was 824/mm^2^, and the PD-L1 immune cell proportion score (the percentage of PD-L1–positive immune cells occupying the tumor) was 40% but the PD-L1 tumor proportion score was 0% (PD-L1 combined positive score was 1–9%). The boxed regions of the upper panels are shown at higher magnification in the lower panels. Scale bars, 100 µm. (**c**) Computed tomography scans showing tumor regression after nivolumab treatment. Left, middle, and right panels correspond to 22 weeks before, 2 weeks before, and 4 weeks after the start of nivolumab administration, respectively. The tumor burden increased during platinum-doublet chemotherapy (from left to middle), but it decreased with nivolumab treatment (from middle to right). Red arrows indicate the tumor lesion. Scale bars, 1 cm. CUP, cancer of unknown primary site.
